# Investigating mosquito net durability for malaria control in Tanzania - attrition, bioefficacy, chemistry, degradation and insecticide resistance (ABCDR): study protocol

**DOI:** 10.1186/1471-2458-14-1266

**Published:** 2014-12-13

**Authors:** Lena M Lorenz, Hans J Overgaard, Dennis J Massue, Zawadi D Mageni, John Bradley, Jason D Moore, Renata Mandike, Karen Kramer, William Kisinza, Sarah J Moore

**Affiliations:** London School of Hygiene & Tropical Medicine, Keppel Street, London, WC1E 7HT U.K; Norwegian University of Life Sciences, P.O. Box 5003, Ås, 1432 Norway; Institut de Recherche pour le Développement (IRD), Maladies Infectieuses et Vecteurs, Ecologie, Génétique, Evolution et Contrôle, Montpellier, Cedex 5, France; Department of Entomology, Kasetsart University, Bangkok, Thailand; Swiss Tropical & Public Health Institute, Socinstrasse, 57, Basel, CH-4002 Switzerland; National Institute for Medical Research, Amani Research Centre, P.O. Box 81, Muheza, Tanzania; Ifakara Health Institute, P.O. Box 74, Bagamoyo, Tanzania; National Malaria Control Program, Ministry of Health and Social Welfare, Dar es Salaam, Tanzania; University of Basel, Petersplatz 1, Basel, 4003 Switzerland

**Keywords:** Long-lasting insecticidal nets, LLINs, Durability, Mosquito net, Hole index, Biological efficacy, Malaria control, Anopheles, Semi-field, Insecticide resistance

## Abstract

**Background:**

Long-Lasting Insecticidal Nets (LLINs) are one of the major malaria vector control tools, with most countries adopting free or subsidised universal coverage campaigns of populations at-risk from malaria. It is essential to understand LLIN durability so that public health policy makers can select the most cost effective nets that last for the longest time, and estimate the optimal timing of repeated distribution campaigns. However, there is limited knowledge from few countries of the durability of LLINs under user conditions.

**Methods/Design:**

This study investigates LLIN durability in eight districts of Tanzania, selected for their demographic, geographic and ecological representativeness of the country as a whole. We use a two-stage approach: First, LLINs from recent national net campaigns will be evaluated retrospectively in 3,420 households. Those households will receive one of three leading LLIN products at random (Olyset^®^, PermaNet^®^2.0 or Netprotect^®^) and will be followed up for three years in a prospective study to compare their performance under user conditions. LLIN durability will be evaluated by measuring Attrition (the rate at which nets are discarded by households), Bioefficacy (the insecticidal efficacy of the nets measured by knock-down and mortality of mosquitoes), Chemical content (g/kg of insecticide available in net fibres) and physical Degradation (size and location of holes). In addition, we will extend the current national mosquito insecticide Resistance monitoring program to additional districts and use these data sets to provide GIS maps for use in health surveillance and decision making by the National Malaria Control Program (NMCP).

**Discussion:**

The data will be of importance to policy makers and vector control specialists both in Tanzania and the SSA region to inform best practice for the maintenance of high and cost-effective coverage and to maximise current health gains in malaria control.

**Electronic supplementary material:**

The online version of this article (doi:10.1186/1471-2458-14-1266) contains supplementary material, which is available to authorized users.

## Background

The recent successes in malaria control in sub-Saharan Africa (SSA), specifically in Tanzania where malaria deaths have reduced by 70% since 2003, has been largely attributable to the massive scale up of vector control tools, particularly Long Lasting Insecticidal Nets (LLINs) [1–3]. However, sustained malaria control is costly, and dependent on continuing political and donor support. As political commitment diminishes, the deliveries of life-saving control tools will slow down and risk the reversal of the huge achievements to date. Global commitments for malaria control in 2012 were approximately US$2.5 billion, far below the estimated sum of US$5.1 billion required for the task [4]. Global funding mechanisms are projected to decelerate even further in the coming years, leaving gaps of US$2.25 billion before achieving universal access to malaria interventions [1]. Therefore, maximising the impact of interventions through selection of the most cost effective and long lasting interventions is a health policy priority.

Despite the huge financial and logistical investments in the development, production and distribution of LLINs worldwide, there are still limited data available on the LLIN durability under user conditions. The World Health Organization (WHO) released specific guidance on LLIN durability monitoring [5, 6], which was incorporated into guidelines for laboratory and field-testing of LLINs [7] to support national governments with the design of standardised net monitoring and evaluation studies. Effective net life has been estimated to be 3-5 years [8], but some studies indicate that LLIN brands may last less than three years under operational conditions [9–12]. It is only recently that researchers have started to investigate net attrition, i.e. how long nets remain in use in a household, and constructed net survivorship curves [5, 13]. Durability of mosquito nets should thus be defined and measured by the whole process of net loss – from attrition and physical damage to the chemical loss of insecticide residue [5].

Net deterioration differs greatly between regions or cultures as care and repair behaviours, maintenance and net use vary from place to place. Thus, nation-wide evaluations of LLINs are required and called for by the WHO [6, 14]. Evaluation of PermaNet^®^2.0 retrieved from six countries [15] and Olyset^®^ nets from seven countries [16] show large between-country variability of LLIN durability. Net products also vary in material, insecticide, or fibre impregnation technology. Such variations are still largely unknown and direct comparisons within sites are scarce [17] (but see [9, 11, 18]). Reliable data need to be collected by National Malaria Control Programs (NMCPs) to inform national procurement decisions for 1) selection of the most suitable net to plan timely replacement, 2) to understand factors associated with net durability to guide behaviour change communication including care and repair interventions, and 3) to assist industry in product improvement. NMCPs need to understand LLIN durability in their local settings because replacing nets too late puts people at risk of disease, but replacing them too often wastes limited resources.

Also, the dramatic increase in pyrethroid resistance in mosquitoes throughout SSA, including Tanzania [19], might be posing a threat to the sustainability of insecticidal control methods [20, 21]. A surveillance system to monitor emerging insecticide resistance, for example using Geographical Information Systems (GIS) [22, 23], would allow governments and national malaria control programmes to plan resistance control strategies [24]. Spatio-temporal analysis of malaria transmission to identify persistent transmission hotspots may maximise cost- and health-effectiveness of control programmes [23]. The determinants and risk factors for net loss and effectiveness vary spatially, but there is a lack of information of which factors play a role in the attrition and deterioration of LLINs.

**Figure 1:**
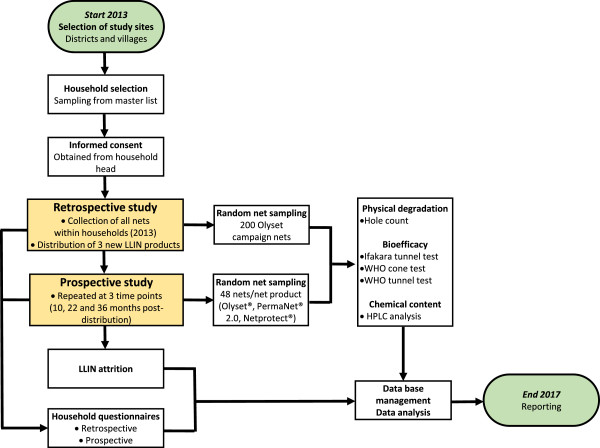
**Flow chart of retrospective and prospective data collection.** After study district and village selection, 45 households will be randomly selected from each village for study inclusion. After obtaining national ethical approval, community sensitisation meetings will be held and written informed consent will be obtained from each household head participating in the study. In the retrospective study in 2013, all nets from study households will be removed after an in-depth household questionnaire and 200 Olyset campaign nets will be sub-sampled for laboratory analysis of LLIN durability components (Table 1). All collected nets will be replaced with one of three new LLIN products; Olyset^®^, PermaNet^®^2.0 and Netprotect^®^ (Table 2) for the prospective study. Ten, 22 and 36 months post-distribution, every household will be followed up with a questionnaire and the presence of each study net will be affirmed to obtain attrition estimates. A random sub-sample of 48 nets per net product will be collected for subsequent analyses consisting of Ifakara Tunnel Tests, hole counts, biological efficacy tests and HPLC analysis.

Therefore, the current study is conducted in collaboration with the Tanzanian NMCP to inform their procurement decisions. The study will be conducted in eight districts in Tanzania, selected for their demographic, geographic and ecological representativeness of the country as a whole. There will be an initial retrospective evaluation of Olyset^®^ nets distributed by the NMCP two-to-four years previously as part of both a targeted and a universal coverage campaign [25]. The same sampled households will then receive one of three LLIN products (Olyset^®^ with the new knit pattern to improve fabric strength, PermaNet^®^2.0 or Netprotect^®^) by random allocation for a prospective follow up study. Effective life of the nets will be assessed at regular intervals for three years using the WHO-recommended set of net durability variables [5] (Table 1) and a set of new methodologies (Figure 1). We will also monitor insecticide resistance in mosquito vectors as an additional component for evaluating LLIN effectiveness to contribute to the growing knowledge within Tanzania, which will assist the NMCP on rational selection of insecticides for vector control. Spatial risk factors of insecticide resistance and LLIN durability, such as land use patterns, agriculture, altitude or distance to potential breeding sites, will be assessed to determine their usefulness in selecting appropriate malaria control strategies by identifying areas where a particular LLIN intervention may be more effective than another.

**Table 1:** **LLIN durability components**

Component	Definition	Response variables for analysis
**A**ttrition	Net loss from household through discarding or use for alternative purpose	- Net presence.
**B**iological efficacy	Ability of net to incapacitate or kill anopheline mosquitoes after contact with insecticide	- Mosquito knockdown (%) 60 minutes post-exposure.
		- Mosquito mortality (%) 24 hours post-exposure.
		- Percentage of bloodfed mosquitoes.
**C**hemical residue	Amount of active ingredient in fibres	- Proportion of nets with active ingredient equal to WHO standard g/kg
**P**hysical degradation	Physical state of the net defined through number, size and location of holes to estimate protection against mosquito bites	- Proportionate Hole Index (pHI) / hole area by location on net.
		- Proportion of nets with a pHI exceeding ≥643 [6].

## Methods/Design

### Study population

**Figure 2:**
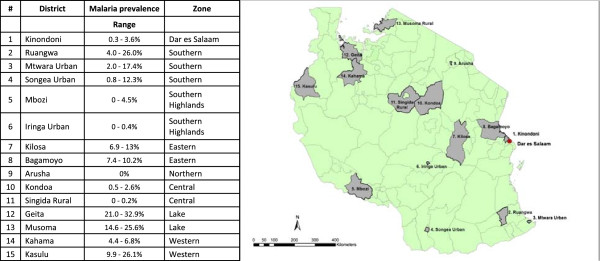
**ABCDR study districts in Tanzania.** Districts selected for LLIN durability (ABCD-components) and the insecticide resistance part of the project (R-component) with malaria prevalence data (% of children aged 6-59 months diagnosed with malaria by Rapid Diagnostic Test and microscopy [29]) and geographical zone. The following eight districts are included in the LLIN study: Kinondoni (1), Mbozi (5), Iringa Urban (6), Kilosa (7), Bagamoyo (8), Geita (12), Musoma Rural (13) and Kahama (14).

The project will be carried out in eight districts representing five of the eight geographical zones of Tanzania and covering variations in malaria epidemiology and ecology. Fifteen districts, i.e. seven districts in addition to the eight previously mentioned, will be included in the mosquito insecticide resistance part of the project (Figure 2). The 15 districts were selected from the 23 districts enrolled in the population arm of the Sentinel Panel of Districts (SPD), SAmple Vital registration with Verbal autopsY (SAVVY) [26]. The 23 SAVVY districts were selected using two-stage sampling with probability proportional to size (PPS) of districts and villages/Enumeration Areas (EAs) from the 2002 Population and Housing Census dataset [27]. In each of the eight districts (Figure 2), all households within 6-20 villages/EAs were enrolled by the SAVVY programme for national representativeness in 2012/2013. We will select ten SAVVY villages per district based on the proximity to district headquarters, except for Kinondoni (Dar es Salaam) where SAVVY only covered six EAs. Using the SAVVY baseline household information, 45 households per village will be randomly selected using the ‘sample’ function in the statistical software R 3.1.1 [28], giving a total of 3,420 households nationwide. Fifty percent more households will be randomly selected as substitution households to accommodate for non-consent or household head absence. The 3,420 study households will be geo-referenced using Global Positioning System (GPS) points to create a GIS database including data on village and house characteristics, socioeconomic variables, net characteristics, and geographical variables, such as environment, land use and potential mosquito breeding sites.

### LLIN products

All three products (Table 2) that will be tested in the prospective study were recommended by WHO Pesticide Evaluation Scheme (WHOPES) at the point of procurement with full approval of Olyset^®^ [16] and PermaNet^®^2.0 [15], and interim approval of Netprotect^®^ [30]. However, Netprotect^®^ approval was withdrawn in September 2014 [31] and from that point on it was decided to replace all sub-sampled study Netprotect^®^ nets with Olyset^®^. The WHOPES working group recommends that programmes should monitor efficacy and performance of Netprotect^®^ under local conditions to obtain further information about the product [32]. Ten nets of each product will be assessed at baseline to ensure that they meet WHOPES thresholds for bioefficacy against anopheline mosquitoes using WHO cone and tunnel tests and insecticidal content with high-performance liquid chromatography (HPLC) analysis.

**Table 2:** **Characteristics of Olyset** ^**®**^ **, PermaNet** ^**®**^ **2.0 and Netprotect** ^**®**^ **net products distributed in the study**

Product name	Product type	Insecticide concentration	Denier	Manufacturer	WHO approval	Reference
Olyset^®^	Permethrin **incorporated** into polyethylene	780 mg/m^2^	>150 denier	Sumitomo Chemicals	Full	[16]
PermaNet^®^ 2.0	Deltamethrin **coated** on polyester	55 mg/m^2^	100 denier	Vestergaard Frandsen	Full	[15]
Netprotect^®^	Deltamethrin **incorporated** into polyethylene	68 mg/m^2^	110 denier	BestNet	Withdrawn	[30, 31]

As a consequence of using LLIN products from different materials (Table 2), the nets may be able to be distinguished physically. However, each net type will be rectangular, of the same dimensions (190 cm x 180 cm x 150 cm) and colour (white) with six loops per net to prevent household participants, technical staff and field team from knowing the treatment allocation as much as possible. A waterproof unique identifying barcode and a five-digit serial number will be attached to each distributed LLIN with a self-laminating laser tag to a hanging loop of the net. This will allow tracking of the nets once they are distributed. The field team will record the net serial number on the questionnaire as the net is distributed to allow the matching up of household and unique net identifying numbers on the net master list.

### Study design

The general study design is shown in a flow chart in Figure 1. One week before the start of the study, a sensitisation meeting will be set up at the district level to inform community leaders (Mwenyekiti and Viongozi), key informants, District Executive Directors (DEDs) and District Medical Officers (DMOs) of the purpose and design of the study. Their permission to work within the community will be sought to inform the community members of the study’s objectives and methods.

#### Retrospective study

Households will be enrolled on written informed consent (Additional file 1). Participants’ houses and questionnaires will be identified by barcodes associated with numeric codes (six-digit serial numbers) to ensure their anonymity and due care will be taken to ensure that only barcodes and numeric codes are used on LLINs and questionnaires, thus blinding participants and researchers to treatment allocation. All the nets from the participating households will be collected and replaced with one of the three new LLIN products (Table 2) chosen at random. The prospective LLIN products will only be known to field teams as net types 1, 2 and 3, thereby blinding and randomising the treatment distribution as much as possible. Each day the field team will receive a household list and a randomly mixed bundle of five sets of type 1, 2 and 3 nets (three nets of same type per set bagged for one household, assuming an average of three sleeping spaces per household). The interviewer will randomly pick one set from the bundle to be distributed when they arrive at each household (modified lottery method). If the household contains more than three sleeping spaces, more nets of the same type will be provided. The interviewer will record the five-digit serial numbers attached to the nets on the questionnaire as described above. Thus, randomisation is conducted by the field workers at the household level, resulting in 15 households per village receiving sufficient nets of one product to cover each sleeping space (Table 3).

**Table 3:** **Households allocated to each net product per village and district in the prospective study**

	Olyset ^®^	PermaNet ^®^2.0	Netprotect ^®^	Total
Districts	8	8	8	8
Villages per district*	10	10	10	10
Households per village	15	15	15	45
Total households	1,140	1,140	1,140	3,420
Total nets**	3,420	3,420	3,420	10,260

*Ten villages selected per district, except for Kinondoni (Dar es Salaam) district with only 6 villages.

**Assuming an average number of 3 nets per households based on the average number of sleeping spaces in households in Tanzania.

A questionnaire will be conducted in Kiswahili, the local language spoken throughout Tanzania, with household heads, or another adult, by the field team (Additional file 2). Respondents will be asked whether they received nets during two NMCP campaigns in 2009-2011. Nets from the campaigns are identifiable by their light-blue colour and size (single), allowing us to differentiate those nets from the campaign and those that might have come from the private sector or Non-Governmental Organisations (NGOs). We will individually assess every net returned to the storage facilities at Bagamoyo Research and Training Centre (BRTC) for its brand label, colour, size, level of cleanliness, and age of manufacture, if available. From those retrospective nets, 200 Olyset^®^ campaign nets will be randomly selected using the ‘sample’ function in R 3.1.1 for durability testing in the laboratory and semi-field systems (Figure 1; Table 1). All other collected Olyset^®^ nets will be recycled by A to Z Textile Mills Ltd (http://www.azpfl.com/index.php/en/).

#### Prospective study

Attrition, net use and user behaviour (Additional file 2), and physical degradation of study nets will be assessed in every consenting household at three subsequent sampling points (10, 22, and 36 months) after the initial LLIN distribution. All households will be surveyed for attrition and a sub-sample of three nets per household will be assessed for physical degradation. Field interviewers will be trained using an amended version of a recently developed USAID/NetWorks-supported training tool kit to assess the number of different category sized holes under field conditions [33].

At each time point, all nets from 48 randomly selected households for each net product will be taken for sub-sampling to validate the D component (physical degradation) assessment in the laboratory, and for B (biological efficacy against mosquitoes) and C (HPLC analysis) components efficacy testing (Figure 1). These households will be randomly selected stratified by district and LLIN product so that six nets from each district per product are evaluated for BCD components. All sampled nets will be replaced with new nets of the same kind except for Netprotect^®^ nets which will be replaced by Olyset^®^, and the sampled household will be excluded from subsequent sampling rounds.

### ABCDR components

#### Attrition (A component)

Attrition of LLINs is defined as the proportion of LLINs that are no longer in use as mosquito nets to sleep under in the receiving household after a given amount of time. This is commonly due to loss through nets being damaged, discarded, or used for other purposes than sleeping under. Nets that are sold, given away or stolen will be excluded from the attrition analysis following WHO guidelines [6] as they may still be “serviceable”.

Trained field interviewers will perform the field visits of all households selected from the master list and voluntarily participating in the study during the sampling points (retrospective sampling, 10, 22 and 36 months after prospective LLIN distribution). Questionnaire data will be collected using Open Data Kit (ODK) Collect software (http://opendatakit.org/use/collect/) on Android tablet computers (Google Nexus One). Observations by the field workers on presence and absence of distributed nets, the location of the net (hanging or stored away), fabric integrity and the net condition are included in the questionnaire (Additional file 2).

#### Physical degradation (D component)

**Figure 3:**
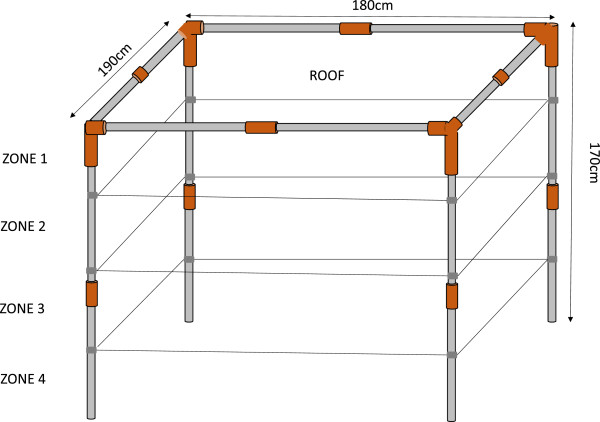
**Mosquito net hole counting by zone.** Collapsible metal frame (170 cm x 180 cm x 190 cm) using locally available materials divided into four 37.5 cm zones from the top to the bottom in order to count holes by zone under laboratory and field conditions.

The physical degradation, or integrity, of the nets will be measured by counting the number, location and size of hole(s) in each net. The proportional hole index (pHI) will be calculated using the hole size categories as per WHO guidelines [5, 6] (Table 4). In addition to the different category sized holes, we will also include five different hole locations on the net by dividing the side panels of the net into a total of four zones from top to bottom, each measuring 37.5 cm, and counting holes in the roof separately as a fifth location (Figure 3). Mosquitoes are more likely to aggregate around certain locations on occupied bed nets (e.g. the roof; [34]). In addition, the lower edges of the bed nets are more likely to be severely damaged, but they are also more likely to be tucked in at night, potentially avoiding mosquito entry [35]. By counting the holes by location, we will be able to take into account these factors when analysing the hole index data and give different weights to holes in different locations. To our knowledge, this formula has not yet been developed. One of our aims is therefore to incorporate hole location into the equation, and to compare its relative importance to a simpler model in terms of protection against mosquitoes in semi-field experiments. Holes will be counted both in the laboratory and in the field using a collapsible metal frame made out of locally available economical materials (Figure 3). In the field, holes in a maximum of three prospective nets will be counted per household due to logistical and time constraints.

**Table 4:** **Hole size categories and their proportionate weights**

Hole category	Hole size description	Hole size (cm)	Hole diameter (cm)	Weight ^a^
**Size 1**	Smaller than a thumb (finger)	0.5-2	1.25	1
**Size 2**	Larger than a thumb but smaller than fist (hand)	2-10	6	23
**Size 3**	Larger than a fist but smaller than a head (head)	10-25	17.5	196
**Size 4**	Larger than a head	>25	30 (assumed)	578

^a^Area of hole divided by 1.23 [6].

#### Biological efficacy (B Component)

Testing will be performed at BRTC, Bagamoyo, Tanzania using *An. gambiae* sensu stricto (s.s.) (Ifakara strain, Njage 1996) mosquitoes that are fully susceptible to insecticides and are reared according to CDC guidelines [36]. Mosquitoes used for testing will be 2-8 days old (depending on the test), nulliparous sugar fed females. Standard WHO cone bioassays will be carried out to evaluate new nets at baseline (ten samples per net product), 200 retrospective Olyset^®^ nets, and a random sub-sample of 48 prospective nets per time point. WHO tunnel tests will be performed if nets fail the cone test [7, 37]. To validate these WHO recommended bioassays and help to estimate fully the protection provided by nets under user conditions, those 48 nets will first be tested in a semi-field tunnel (SFT) – the newly developed Ifakara Tunnel Test (ITT) - to measure the protective efficacy of the nets to people resting underneath them [38]. For the WHO tunnel test and ITT, only those mosquitoes that are responsive to human odour on the day of testing will be used. For semi-field tests, mosquitoes will be deprived of sugar solution for six hours prior to experiments and transferred to a screened test cage one hour prior to testing to allow them to acclimatise.

##### Ifakara tunnel test (ITT)

A semi-field enclosure is here defined as an enclosed environment, ideally situated within the natural ecosystem of the target disease vector and exposed to ambient environmental conditions. Semi-field enclosures offer several useful features: 1) participants are safe because they are exposed only to laboratory-reared disease-free mosquitoes, 2) experiments can be run using standard numbers of mosquitoes allowing year round collections regardless of natural vector populations, and 3) using mosquitoes of known age, physiological status and avidity reduces experimental variability allowing for rapid data collection and improved data quality.

**Figure 4:**
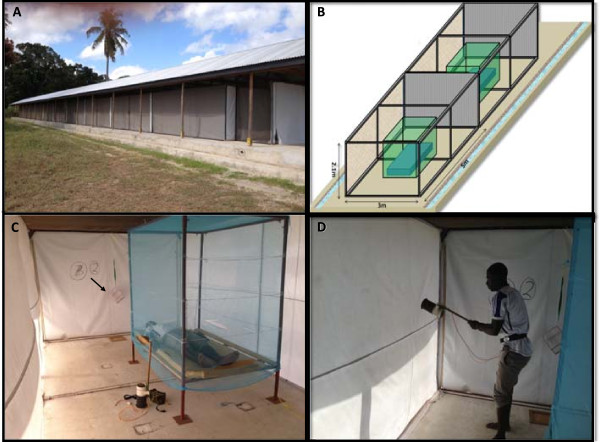
**Experimental set-up in the Ifakara Tunnel Test (ITT).** Ifakara Tunnel situated at Bagamoyo Research Training Centre (BRTC) in Kingani, Bagamoyo. The tunnel is a 50 m long, 3 m wide and 2.1 m high steel tube frame construction upon a concrete base surrounded by a water channel and beneath a beamed wooden frame supporting a corrugated steel roof **(A)**. Net covered tunnel divided into 10 individual compartments containing bed and adjustable steel net frame **(B)**. Experimental set up for Ifakara Tunnel Test. A human volunteer will sleep beneath the LLIN from 21.00 hrs to 06.00 hrs. *Anopheles gambiae* mosquitoes will be released from the holding cages by raising a cage from its wooden base (arrow) using a nylon string **(C)**. At the end of the experiment, all mosquitoes within each of the compartments will be removed with a Prokopack **(D)**.

The Ifakara tunnel is a 50 m long, 3 m wide and 2.1 m high steel tube frame construction covered by durable UV resistant polyurethane coated netting (Figure 4A). The structure is constructed upon a concrete base surrounded by a water channel to prevent entry by ants and spiders. The tunnel sits beneath a simple beamed wooden frame supporting a corrugated steel roof to allow work in all weather conditions. The netted tunnel is divided into ten individual test chambers (5 m x 3 m x 2.1 m) with interconnecting doors that are sealed with zips and Velcro to prevent mosquitoes moving from one test chamber to another (Figure 4B). Each end of the tunnel contains an additional double door module to prevent loss of laboratory-reared mosquitoes into the wild. Mosquitoes will be released within each compartment by raising the netted holding cages from their removable wooden bases. This is achieved remotely by the volunteer in each compartment pulling a nylon cord to raise the cage whilst remaining beneath the net (Figure 4C). After the allotted experimental time period, all mosquitoes within each of the compartments will be removed by mechanical aspiration (Figure 4D).

Each of the ten experimental compartments will be provided with a steel bed frame and foam mattress upon which a volunteer will sleep during each test and over which the LLIN will be draped (Figure 4C). A human volunteer will sleep beneath the LLIN from 21.00 hrs to 06.00 hrs to represent user conditions. For each test, 30 nulliparous 2-8 day old, disease-free *An. gambiae* s.s. mosquitoes will be introduced. At 06.00 hrs, the mosquitoes within the compartment will be collected using a mechanical aspirator (Prokopack; [39]) and scored for knockdown (KD), 24-hour mortality and blood-feeding success.

All participants in ITT experiments will be male staff members of IHI who have received appropriate training and are experienced in conducting semi-field tunnel tests. All participants will be recruited on written informed consent, which explains the risks and benefits of the study and are free to leave the study without explanation. The risk of disease transmission to volunteers is very low.

#### Chemical residue (C Component)

After biological efficacy and physical degradation testing in semi-field facilities in Bagamoyo has taken place, the same 48 LLINs per product will be used for chemical residue analysis. Chemical residues will be determined by HPLC [40]. The HPLC analysis will be carried out in a WHO Collaborating Centre for Quality Control of Pesticides (Walloon Agricultural Research Centre; CRA-W) following the latest WHO recommendations. Four sub-samples of 30 cm x 30 cm will be taken from each net representing the entire net. Samples will be kept at 4°C in aluminium foil until analysed to determine the total content of permethrin (Olyset^®^) or deltamethrin (Netprotect^®^ and PermaNet^®^2.0) in g/kg.

#### Resistance monitoring (R Component)

The resistance monitoring component builds upon the existing nationwide longitudinal monitoring of insecticide resistance in Tanzania that has already been carried out in 26 selected sentinel districts from different ecological zones of Tanzania [41]. In the current study, insecticide resistance will be assessed in a total of 15 districts (Figure 2). Eight of these districts coincide with the ABCD part of the project. Insecticide resistance will be monitored in cross-sectional countrywide surveys conducted annually throughout the project life. These surveys will be carried out in May/June, just after the long rainy season. The susceptibility levels and resistance mechanisms of malaria vectors to insecticides of public health and agricultural relevance in Tanzania will be determined. Results will feed into the online geospatial application IR Mapper [23]. *Anopheles* larvae will be collected in easily accessible larval habitats in one or two villages per district. Each breeding site will be geo-referenced using GPS. Larvae will be bred to adult mosquitoes in field laboratories, which will be maintained on 10% glucose solution in mosquito cages. Three- to five-day old F1 generation mosquitoes will be tested using standard WHO insecticide susceptibility testing procedures [42]. Mosquitoes will be exposed to papers impregnated with the WHO-recommended discriminating concentrations (v⁄w) of 0.05% deltamethrin, 0.05% lambda-cyhalothrin, 0.75% permethrin, 0.1% bendiocarb, 1% fenitrothion and 4% DDT prepared at University Sains, Malaysia [42]. During exposure, KD rates will be recorded after a range of exposure times. Mosquitoes will then be provided access to 10% glucose solution and 24 hour mortality will be scored. All mosquitoes will be identified using keys described by Gillies [43, 44] and *An. gambiae* sibling species identified using established Polymerase Chain Reaction (PCR)-based methods [45]. PCR-based standard methods will also be used to detect kdr mutations [46] and biochemical assays will be used to detect the enzyme-based resistance mechanisms in mosquitoes.

### Statistics and data analysis

#### Sample size calculation

Sample size calculations were based on the primary outcome measure of net attrition using the standard formula for the difference between two proportions [47]. The BCD components were treated as an additional sub-sample to the original calculated sample size. Assuming an average of 3 nets per household and a coefficient of variation of 0.25, then the formula on page 110 of Hayes & Moulton [48] gives a sample size of 973 households per arm to detect a difference in attrition between two brands with attrition rates of 47.5% and 52.5% with 90% power. Therefore, there will be at least 90% power to detect a 5% difference in attrition rates. Loss to follow up and households excluded due to sub-sampling have been added to the final sample size to give (1,140 households * 3 nets/ household) = 3,420 LLINs per LLIN product (Table 3).

#### Data analysis

We will collect a set of response variables (Table 1) and explanatory variables. The explanatory variables will be collected from household questionnaires and observations and will include time after net distribution, net product, geographical location, patterns of net use (e.g. type of bed, frequency of net use), net status, washing and handling, perceptions of nets and socioeconomic status of the household. All response variables will be analysed using the statistical programs STATA^®^13 (http://www.stata.com/) and R (http://www.R-project.org/). Regression modelling including multivariate generalised linear models and generalised linear mixed models will be used to determine covariates affecting net durability components such as LLIN age, geographical location and data collected from household surveys. Principal Component Analysis (PCA) will be used to determine a combination of variables for socioeconomic status to explain the overall observed variation and reduce the complexity of the data. In order to analyse net attrition and physical degradation in more detail, 95% confidence intervals will be calculated for the attrition and ‘unserviceable’ physical condition of each net product at the three prospective time points. At each point, logistic regression with a category for each brand of net will be performed to assess if there is a difference in attrition between the three net products. If a significant difference is found, then pairwise comparisons will be examined.

### Ethical considerations

Full ethical approval has been obtained from ethical review committees at London School of Hygiene & Tropical Medicine (6333/A443), Ifakara Health Institute (IHI/IRB/AMM/ No: 07- 2014) and the National Institute for Medical Research (NIMR/HQ/R.8c/Vol. I/285).

Written informed consent will be obtained from the head of the household of those households selected for participation (Additional file 1). If absent, another adult household member (above the age of 18) representing the household head will sign the informed consent form. The informed consent will be obtained before each survey. For participants who cannot read the form, the informed consent form will be read out and explained by the local field staff in Kiswahili or the local language in the presence of a community witness. After consenting, the household head, or his representative, will be asked to mark a thumb impression on the form, and the witness will be asked to sign it. The potential participants will be advised that they can refuse to participate at any point in the future and may still keep their new net.

## Discussion

In addition to following WHO durability guidelines [5], which will allow direct comparison between our study and other ongoing durability investigations in SSA, we are also developing new methodologies to fully assess to what extent physical degradation, chemical decay and biological efficacy actually determine the life of a net, i.e. the duration of its effective protection. LLINs act as a barrier against blood-feeding of anopheline mosquitoes on humans. We will determine the effectiveness of nets as transmission barriers by testing the whole net from the field protecting humans throughout the night against mosquito bites in semi-field Ifakara Tunnel Tests (ITT). This will give us a strong measure of the individual protective efficacy against human biting behaviour. In addition, it will allow us to estimate the mortality of mosquitoes exposed to LLINs under more natural conditions, a methodology that is commonly performed in experimental huts [49]. However, the ITT is designed to increase both data throughput and data power because it evaluates eight nets and two controls per night using mosquitoes of identical physiological status. In addition, the same number of mosquitoes can be released into each of the compartments so that the effect of the efficacy of the nets is measured in the same way in each compartment. In contrast, field tests require far greater numbers of replicates to achieve good statistical power due to both spatial and temporal heterogeneities in mosquito numbers [50]. We will also determine the WHO-recommended hole index (pHI) by location on the net, with the potential of influencing further net product design with strengthened material in the bottom quarter of the net.

National and international public health policy makers may therefore use the information provided by this, and other ongoing studies, to procure the most cost- and health-effective nets. Results will allow the selection of nets that provide protection against disease at optimum costs (trading off LLIN durability, price and insecticide resistance status of local mosquito populations), and to estimate the timing of repeated distribution campaigns to ensure that maximal health gains are maintained.

## Current study status

At the time of submission of this manuscript (December 2014), the study had completed the retrospective data collection and random distribution of the three new LLIN products, the establishment of the return net data base, and the prospective household survey after 10 months.

## Electronic supplementary material

Additional file 1: **English version of the informed consent form that will be used to obtain written informed consent from heads of household in the retrospective study.** This informed consent form has been translated into Kiswahili. (PDF 336 KB)

Additional file 2: **English version of the prospective questionnaire that will be programmed in Kiswahili using ODK Collect on Google Nexus tablet computers to collect basic household and net attrition and use information.** (PDF 517 KB)
