# Supplementary material for: Investigating mosquito net durability for malaria control in Tanzania - attrition, bioefficacy, chemistry, degradation and insecticide resistance (ABCDR): study protocol
Source: BMC Public Health. 2014 Dec 13;14:1266. doi: 10.1186/1471-2458-14-1266 (PMC4301422; doi:10.1186/1471-2458-14-1266)
Supplement: Supplementary file 1 — Additional file 1: English version of the informed consent form that will be used to obtain written informed consent from heads of household in the retrospective study. This informed consent form has been translated into Kiswahili. (PDF 336 KB) [file 12889_2014_7451_MOESM1_ESM.pdf]

INFORMED CONSENT FORM – Survey

<u>Name of project:</u> The useful life of bednets for malaria control in Tanzania

Part 1. Information sheet

My name is <________________________> and I work for Ifakara Health Institute.

Malaria is still a problem in Tanzania and we want to work with you to find ways to stop malaria. Malaria is transmitted by the bite of an infected mosquito that bites after sunset. A good way to stop malaria is to use a strong bednet that has insecticide to kill mosquitoes that try to bite while you are sleeping. Therefore, in 2009 and 2010, the government of Tanzania provided everyone in the country with a strong bednet. These bednets have been shown to last for three years without getting many holes and we want to make sure that bednets are still working to kill mosquitoes after this time in all the areas of Tanzania so we can tell the government if the nets are still good and helping to stop malaria.

We first discussed this project with the mwenyekiti and he decided that we were allowed to work in this village. Then, we drew lots from the list of all households in the village. Your house is one of those that was chosen. If you agree, we would like to ask you some questions about your household and to see your bednets to see if they are still good enough to use, how many holes they have and what you think about the bednets you were given.

We will take all your old bednets and then we will give you a new bednet for each sleeping space in your household. We will put a number on the wall of your house so that we know when we came to visit you and then we might come back to your house again at least one more time some time in the next three years to ask you some more questions about the new bednets that we have given you. We will draw lots again to see which households we will visit.

Risks

There is no risk in participating in this study. All the bednets that we will give to replace your old nets have been approved for use by the government of Tanzania. You must make sure you follow the instructions on the packet to make sure the bednets works very well to kill mosquitoes and we will talk to you about the instructions if you have any questions.

Benefits

You will receive a new bednet for each sleeping space in your house at no cost to stop getting mosquito bites so you will be safer from malaria. You will also be helping the government of Tanzania provide its citizens with better healthcare because all the information we collect will be used to help the government buy the best bednets and replace them when it is necessary to make sure everyone is protected from malaria.

Your participation is voluntary. You can look at the list of questions and you can refuse to answer any questions if you do not want to answer it, and you can choose to stop at any time. All of the answers are secret because we will not put your name on the questionnaire, we will use a number and initials of each household member instead. If you decide that you do not want to continue with the study then you are free to stop taking part at any time. The questions will take about 45 minutes and will help us understand more about how many years the bednets last to stop mosquito bites in this area of Tanzania. I will also need to see the bednet, so I can see how much it has worn. You can talk to a relative before you decide to take part in the study if you want to.

If you have any questions about this study at any time, contact Dennis Massue the project leader at NIMR (Telephone: [redacted]) or Mwifadi Mrisho a research scientist at IHI (Telephone [redacted]).

Informed consent record for the participant

I clearly understand the aims of the project entitled "Useful life of bednets in Tanzania". I agree for myself and all the people in my family to take part in the study. I understand that

1) I will be asked some questions about my household and will give all my bednets to the project

2) I can choose not to answer any question if I don't want to answer

3) I will be given a new bednet for every sleeping space in my house

4) Someone from the project might visit my house again some time in the next three years and ask me some more questions about the bednets I have been given

We will give you a copy of this consent form to keep.

Participant Name: ____

Participant Signature: ______________________Date_________________

Witness Name: ____

Witness signature: _______________________________Date_________________

Household identification No.: _____________

Interviewer Identification No.: _____________

Investigators: Dr Hans J Overgaard; Dr William Kisinza; Dr Renata Mandike; Dr Sarah J Moore; Dr Lena M Lorenz
Organizations: Ifakara Health Institute (IHI), National Institute of Medical Research (NIMR), London School of Hygiene & Tropical Medicine (LSHTM), University of Life Sciences Norway (UMB)
