# Supplementary material for: Investigating mosquito net durability for malaria control in Tanzania - attrition, bioefficacy, chemistry, degradation and insecticide resistance (ABCDR): study protocol
Source: BMC Public Health. 2014 Dec 13;14:1266. doi: 10.1186/1471-2458-14-1266 (PMC4301422; doi:10.1186/1471-2458-14-1266)
Supplement: Supplementary file 2 — Additional file 2: English version of the prospective questionnaire that will be programmed in Kiswahili using ODK Collect on Google Nexus tablet computers to collect basic household and net attrition and use information. (PDF 517 KB) [file 12889_2014_7451_MOESM2_ESM.pdf]

PROSPECTIVE HOUSEHOLD QUESTIONNAIRE

"The useful life of bednets for malaria control in Tanzania: Attrition, Bioefficacy, Chemistry, Durability and insecticide Resistance"

Introduction: Hello, my name is "………..". I am from IHI and work on a project investigating how long bed nets last in Tanzania. Maybe you remember my team from last year when we visited.

To be filled in before the interview

0.0 Household Identification number |____|____|____|____|____|____|

0.1 Repeat Household Identification number |____|____|____|____|____|____|

0.2 Code of interviewer |____|____|

0.3 Date of interview |____|____| / |____|____| / |____|____|____|____| (Day/Month/Year)

0.4 Name of district |____|

0.5 Name of village |____|

0.6 GPS coordinates of household: S: |__|__|__|__|__|__|__| E |__|__|__|__|__|__|__|

0.7 Is this the same family that was visited last year?
[__] Yes
[__] No – STOP

0.8 INFORMED CONSENT OBTAINED:
[__] Yes
[__] No – STOP

Section 1: *"I would like to ask you (head of household or adult > 18 years) some questions about your household"*

Section 1.1: Household listings

"I would first like to ask you some information about the members of your household and any temporary visitors to your household."

Nr	First name (residents + visitors)	Relationship to head of household?	Gender (1…Male, 2…Female)	Age (years), if less than 1 year: 00	Age (months) if less than 1 year	Highest level of education	Usual resident or temporary visitor?	Currently pregnant? (01…Yes, 00…No, 99…Don't know/NA)	Slept here last night? (1…Yes, 0…No)
1.1	1.2	1.3 - code	1.4	1.5	1.6	1.7 - code	1.8 - code	1.9	1.10
01		|__|__|	|__|	|__|__|	|__|__|	|__|__|	|__|	|__|__|	|__|
02		|__|__|	|__|	|__|__|	|__|__|	|__|__|	|__|	|__|__|	|__|
03		|__|__|	|__|	|__|__|	|__|__|	|__|__|	|__|	|__|__|	|__|
04		|__|__|	|__|	|__|__|	|__|__|	|__|__|	|__|	|__|__|	|__|
05		|__|__|	|__|	|__|__|	|__|__|	|__|__|	|__|	|__|__|	|__|
06		|__|__|	|__|	|__|__|	|__|__|	|__|__|	|__|	|__|__|	|__|
07		|__|__|	|__|	|__|__|	|__|__|	|__|__|	|__|	|__|__|	|__|
08		|__|__|	|__|	|__|__|	|__|__|	|__|__|	|__|	|__|__|	|__|
09		|__|__|	|__|	|__|__|	|__|__|	|__|__|	|__|	|__|__|	|__|
10		|__|__|	|__|	|__|__|	|__|__|	|__|__|	|__|	|__|__|	|__|
11		|__|__|	|__|	|__|__|	|__|__|	|__|__|	|__|	|__|__|	|__|
12		|__|__|	|__|	|__|__|	|__|__|	|__|__|	|__|	|__|__|	|__|
13		|__|__|	|__|	|__|__|	|__|__|	|__|__|	|__|	|__|__|	|__|
14		|__|__|	|__|	|__|__|	|__|__|	|__|__|	|__|	|__|__|	|__|

Codes for relationship to head of household (1.3):	Codes for highest level of education (1.7):	Codes for usual resident or visitor (1.8):
01…Head of household<br>02…Spouse<br>03…Son or daughter<br>04…Son-in-law or daughter-in-law<br>05…Grandchild<br>06…Parent<br>07…Parent-in-law<br>08…Brother or sister<br>09…Nice or nephew<br>10…Other relative<br>11…Adopted/foster/stepchild<br>12…Not related	01…Never attended school<br>02…Some primary school<br>03...Completed primary school (grade 7)<br>04…Some secondary school<br>05…Completed secondary school O-level (Form 4)<br>06… Completed secondary school A-level (Form 6)<br>07…Higher education (university/college/vocational training)<br>99…Don't know	1…Usual resident<br>2…Temporary visitor

"Just to make sure that I have a complete listing, are there any other persons living in your household that we have not listed, such as small children or infants?"

- *Go through list with respondent*
- *If yes, add these individuals to table above*

"Are there any other people living or staying here who may not be members of your family, such as visitors or friends or temporary workers?"

- *If yes, add these individuals to table above*

Section 1.2: Household characteristics

"Now I would like to ask you some general questions about this household."

Q #	Questions and filters	Coding category	Answer (enter coding categories)
1.11	Who is responding to the questions?	01…Head of household<br>02…Partner of household head<br>03…Other adult in household, specify	|__|__| ______________________
1.12	How old is the respondent?	***Age in years***	|__|__| if less than 18, STOP
1.13	What is the main source of income in the household head?	01…Salary<br>02…Business<br>03…Farming/livestock keeping<br>04…Skilled labour/Entrepreneurship (fundi, tailor)<br>05…Casual labour (kibarua)<br>06…Fishing<br>07…Driver/taxi/bajaji<br>08…Student<br>09…Pension<br>10…No source of income<br>11…Other, specify	|__|__| ______________________
1.14	What is the main material of the roof?<br>***Observe***	01…Grass /palm thatch<br>02…Corrugated iron sheets<br>03… Other metal, e.g. korie<br>04… Tembe house (roofed with soil)<br>04… Other, specify	|__|__| ______________________
1.15	What is the main material of the walls?<br>***Observe***	01…Mud and sticks<br>02…Burned bricks<br>03…Cement bricks<br>04…Mud bricks (Matofali mabichi)<br>05…Other, specify	|__|__| ______________________
1.16	What is the main material of the floor?<br>***Observe***	01…Earth<br>02…Cement<br>03…Tiles<br>04…Carpet<br>05…Wood<br>06…Other, specify	|__|__| ______________________
1.17	Are any of the windows screened with netting?<br>***Observe***	01…Yes<br>00…No, **go to 1.18**	|__|__|
1.17a	What are the windows screened with?<br>***Observe***	01…Wire mesh (metal/plastic)<br>02…Old bednet<br>03…Other material, specify	|__|__| ______________________

1.18	Does the house have an open eave gap?<br>***Observe***	01…Yes<br>00…No		|__|__|
1.19	Does this house have a ceiling?<br>***Observe***	01…Yes<br>00…No		|__|__|
1.20	What type of fuel does your household mainly use for cooking?	01…Electricity<br>02…Gas<br>03… Kerosene<br>04… Diesel-powered generator<br>05…Charcoal<br>06…Firewood/straw<br>07…Other, specify		|__|__| ______________________
1.21	Does your house use any of the following sources of energy?<br><br>***Prompt each category.***	01…Yes<br>00…No	Electricity<br>Hurricane lamp<br>Candle<br>Traditional lamp<br>Fire<br>Battery/solar<br>Generator<br>None<br>Other, specify	|__|__| |__|__| |__|__| |__|__| |__|__| |__|__| |__|__| |__|__| ________________________
1.22	What is the principal type of toilet facility used by members of the household?	01…Own flush toilet<br>02…Shared flush toilet<br>03…Own pit latrine<br>04…Shared pit latrine<br>05…Bush/forest/field		|__|__|
1.23	What is the principal household source of drinking-water?	01…Piped water in home or yard/bottled water<br>02…Rain water collection<br>03…Own well/pump<br>04…Shared well/pump<br>05…River/stream/pond/lake<br>06…Water truck/cart<br>07…Other, specify		|__|__|
1.24	Does your household possess any of the following items?<br><br>***Prompt each category***	01…Yes<br>00…No	Mobile phone<br>Radio<br>Refrigerator/freezer<br>Electric Fan<br>Television	|__|__| |__|__| |__|__| |__|__| |__|__|

			Satellite Dish/Cable<br>Generator<br>Air conditioner<br>None of the above	|__|__| |__|__| |__|__| |__|__|
1.25	Does the household (any member) have any of the following means of transport?<br><br>***Prompt each category***	01…Yes<br>00…No	Bicycle<br>Motorbike<br>Car/Truck<br>Bajaji<br>Animal/Cart<br>Boat/Canoe/Jahazi<br>None of the above	|__|__| |__|__| |__|__| |__|__| |__|__| |__|__| |__|__|
**Section 2: *"Now I would like to ask you some questions about your bednets."***				
2.1	How many sleeping places are there in your household?<br>***Include all sleeping spaces where a net could be hung up, or has ever been hung up, including if there is more than one sleeping space in each room used for sleeping***			Indoors |__|__| Outdoors |__|__|
2.1a	How many sleeping places <u>were used</u> last night in your household?			Indoors |__|__| Outdoors |__|__|
2.2	How many mosquito nets that can be used for sleeping does your household have in total?<br>***Probe for nets not in use: stored, saved, unopened***			|__|__|

Section 2.1: Prospective roster.

Interviewer to find household list in household folder and enter first net serial number into PDA.

"Now could you please show me the nets in your household. I will need access to the barcode that is attached to the net."

Q #	Questions and filters	Coding Category	Net 1	Net 2	Net 3
2.3	Net serial number<br><br>***(from household list in household folder)***		|_|_|_|_|_|_|	|_|_|_|_|_|_|	|_|_|_|_|_|_|
2.4	Re-enter net serial number<br><br>***…once net is identified.***	***If net is no longer present, re-enter from household list.***	|_|_|_|_|_|_|	|_|_|_|_|_|_|	|_|_|_|_|_|_|

2.5	Net still in possession of the household	01…Yes, **go to 2.7**<br><br>00…No	|__|__|	|__|__|	|__|__|
2.6	If no, why not?	01…Net thrown away **Go to 2.6.1**<br><br>02…Net used for something else **Go to 2.6.5**<br><br>03…Net was given away **Go to 2.6.7**<br><br>04…Net was sold **Go to 2.6.10**<br><br>05…Net was stolen **Go to 2.6.10**<br><br>99…Don't know **Go to 2.6.10**	|__|__|	|__|__|	|__|__|
2.6.1	Why was the net thrown away?	01…Too damaged for sleeping under **Go to 2.6.2**<br><br>02…Did not like the net for sleeping under **Go to 2.6.3**<br><br>99…Don't know **Go to 2.6.4**	|__|__|	|__|__|	|__|__|
2.6.2	How was the net damaged?<br><br>***Do not prompt. Record all reasons that the person mentions.***<br><br>**Go to 2.6.4**	01…By fire<br><br>02…Rodents<br><br>03…Children<br><br>04…Wear and tear<br><br>05…Other, specify<br><br>99…Don't know	|__|__| <br>__________	|__|__| <br>__________	|__|__| <br>_________
2.6.3	Why did you not like the net?<br><br>***Do not prompt. Record all reasons that the person mentions.***<br><br>**Go to 2.6.4**	01…Too hot<br><br>02…Net too small<br><br>03…Net too big<br><br>04…Mesh size too big<br><br>05…Don't like the feel of the material<br><br>06…Don't like the colour<br><br>07…Net too dirty<br><br>08…Don't like the smell<br><br>09…Net makes me sneeze, itch, head ache	|__|__| <br>__________	|__|__| <br>__________	|__|__| <br>_________

		10… Infested with bed bugs<br><br>11… Doesn't protect against mosquitoes<br><br>12…Other, specify<br><br>99…Don't know			
2.6.4	How did you discard of the net?<br><br>**Go to 2.6.10**	01…Burned inside the house<br><br>02…Burned outside the house<br><br>03…Buried<br><br>04…Threw away as rubbish, <u>specify where</u><br><br>05…Recycled<br><br>99…Don't know	|__|__| <br>__________	|__|__| <br>__________	|__|__| <br>_________
2.6.5	Why did you use the net for something else?	01…Too damaged for sleeping under<br><br>02…Did not like the net for sleeping under<br><br>03…More useful things to do with it<br><br>99…Don't know	|__|__|	|__|__|	|__|__|
2.6.6	If used for something else, what was it used for?<br><br>**Go to 2.6.10**	01…Screen windows/doors<br><br>02…Screen or fence toilet<br><br>03…Protect garden (fence in or cover crops from birds)<br><br>04…Protect animals (chickens or ducks)<br><br>05…Fishing<br><br>06…Mattress/pillow<br><br>07…Agriculture, e.g. dry cassava<br><br>08…Make rope<br><br>09…Stored for visitors<br><br>10…Other, specify<br><br>99…Don't know	|__|__| <br>__________	|__|__| <br>__________	|__|__| <br>_________
2.6.7	If given away, to whom?	01…Neighbours<br><br>02…Children going to school/college<br><br>03…Parents	|__|__|	|__|__|	|__|__|

		04…Other relatives<br><br>05…Others, specify<br><br>99…Don't know	________	________	________
2.6.8	If given away, why?	01…Too many nets in household **Go to 2.6.10**<br><br>02…Someone else needed net more **Go to 2.6.10**<br><br>03…Replaced it with a better net **Go to 2.6.9**<br><br>04…I do not like to use nets **Go to 2.6.10**<br><br>05…Other, specify **Go to 2.6.10**<br><br>99…Don't know **Go to 2.6.10**	|__|__| <br>________	|__|__| <br>________	|__|__| <br>________
2.6.9	If replaced by a better net, why did you like the replacement net more?<br><br>***Do not prompt. Record all reasons that the person mentions.***	01…Colour, specify which colour is preferred<br><br>02…Less damaged<br><br>03…Cleaner<br><br>04…More suitable size<br><br>05…More suitable length<br><br>06…Nicer texture / material<br><br>07…It was free<br><br>08… Other, specify<br><br>99…Don't know	|__|__| <br>________	|__|__| <br>________	|__|__| <br>________
2.6.10	When was the net lost from the household?<br><br>**Go to NEXT NET or if there are no more ABCDR nets, to Section 5**	01…less than 1 month ago<br><br>02…between 1 and 3 months ago<br><br>03…between 4 and 6 months ago<br><br>04…more than 6 months ago<br><br>05…more than 1 year ago<br><br>99…Don't know	|__|__|	|__|__|	|__|__|
2.7	Where is the net located?<br><br>***Observe***	01…Hanging loose over a sleeping space<br><br>02…Hanging and folded up or tied<br><br>03…Stored inside a bag<br><br>04…Stored but not in a bag	|__|__| <br>________	|__|__| <br>________	|__|__| <br>________

		05…Other, specify			
2.8	What type of sleeping space is the net used for?	01…Wooden bedframe<br>02…Stick bedframe<br>03…Iron bedframe<br>04…Mattress (no frame)<br>05…Reed mat (no frame)<br>06…Floor<br>07…Never used → **go to 2.8a**<br>08…Other, specify	|__|__| ________	|__|__| ________	|__|__| ________
2.8a	Why has the net never been used?<br>**Go to NEXT NET or if there are no more ABCDR nets, to Section 5**	01...Save the net for visitors<br>02…Save the net for future use<br>03…No place to hang up<br>04…Currently have enough nets in use<br>05…Other, specify	|__|__| ________	|__|__| ________	|__|__| ________
2.9	What is the main material of the roof in this room?<br>***Observe***	01…Grass /palm thatch<br>02…Corrugated iron sheets<br>03… Other metal, e.g. korie<br>04… Tembe house (roofed with soil)<br>04… Other, specify	|__|__| ________	|__|__| ________	|__|__| ________
2.10	What is the main material of the walls in this room?<br>***Observe***	01…Mud and sticks<br>02…Burned bricks<br>03…Cement bricks<br>04…Mud bricks (Matofali Mabichi)<br>05…Other, specify	|__|__| ________	|__|__| ________	|__|__| ________
2.11	What is the main material of the floor in this room?<br>***Observe***	01…Earth<br>02…Cement<br>03…Tiles<br>04…Carpet<br>05…Other, specify	|__|__| ________	|__|__| ________	|__|__| ________

2.12	Was this net used last night?	01…Yes **Go to 2.14**<br><br>00…No	|__|__|	|__|__|	|__|__|
2.13	If not, why not?<br><br>***Do not prompt. Record all reasons that the person mentions.***<br><br>**Go to 2.16**	01…User did not sleep here<br><br>02…Net was not available to use<br><br>03…Used a different net<br><br>04…No malaria now<br><br>05…No mosquitoes<br><br>06…Net too old or too torn<br><br>07…Net is dirty<br><br>08…Net full of bedbugs<br><br>09…Net too hot<br><br>10…Net too small<br><br>11…Net too big<br><br>12…Mesh size too big<br><br>13…Don't like the material<br><br>14…Don't like the colour<br><br>15… Don't like the smell<br><br>16…Net made me sneeze, itch, head ache<br><br>17…Other, specify<br><br>99…Don't know	|__|__| <br>__________	|__|__| <br>__________	|__|__| <br>__________
2.14	Who used this net last night?	***Drop down menu with names from household roster 1.2. Follow up with "Is [name] x years old?"***<br><br>***Allow multiple choices***	__________<br>__________<br>__________<br>__________	__________<br>__________<br>__________<br>__________	__________<br>__________<br>__________<br>__________
2.15	During the previous week, how many times has the net been used?	01…Every night<br><br>02…5-6 nights<br><br>03…1-4 nights<br><br>04…None of the nights	|__|__|	|__|__|	|__|__|

		99…Don't know				
2.16	Do you use any of the following sources for cooking, heating or lighting in the same room as the net?	01…Yes<br>00…No	Firewood<br>Charcoal<br>Gas<br>Hurricane lamp<br>Candle<br>Koroboi<br>Cigarettes	|__|__| |__|__| |__|__| |__|__| |__|__| |__|__| |__|__|	|__|__| |__|__| |__|__| |__|__| |__|__| |__|__| |__|__|	|__|__| |__|__| |__|__| |__|__| |__|__| |__|__| |__|__|
2.17	In the last 6 months, have you seen any rats or mice in this room or their traces (faeces or damage)?	01…Yes<br>00…No<br>99…Don't know		|__|__|	|__|__|	|__|__|
2.18	Do cats have access to this room?	01…Yes<br>00…No<br>99…Don't know		|__|__|	|__|__|	|__|__|
2.19	During which periods of the year is this net used to sleep under?	01…All year<br>02…Rainy season only<br>03…Dry season only<br>99…Don't know		|__|__|	|__|__|	|__|__|
2.20	Is this net ever used for sleeping under away from the main house?	01….Yes<br>00….No, **go to 2.21**<br>99….Don't know, **go to 2.21**		|__|__|	|__|__|	|__|__|
2.20a	Where is the net used for sleeping under away from the main house?	01….Taken to school/college<br>02….Taken to other house<br>03….Taken to the farm hut/forest<br>04….Other, specify		|__|__| __________	|__|__| __________	|__|__| _________
2.21	Do you tuck the net in at night?	01….Yes, **go to 2.22**<br>00….No<br>99….Don't know, **go to 2.22**		|__|__|	|__|__|	|__|__|

2.21a	Why do you not tuck the net in?	01…Net not long enough<br>02…Nothing to tuck under<br>03…Feel too closed in<br>04…Too much effort<br>05…Other, specify	|__|__| __________	|__|__| __________	|__|__| __________
2.22	Has the net ever been washed?	01….Yes<br>00….No, **go to 2.27**<br>99….Don't know, **go to 2.27**	|__|__|	|__|__|	|__|__|
2.22a	How many times did you wash the net in the last year?	01…Once<br>02…Once every 6 months<br>03…Once every 3 months<br>04…Every month<br>99….Don't know	|__|__|	|__|__|	|__|__|
2.23	When was the last time you washed the net?	01…less than 1 month ago<br>02…between 1-3 months ago<br>03…between 4-6 months ago<br>04…between 6-12 months ago<br>05…more than 1 year ago<br>99…Don't know	|__|__|	|__|__|	|__|__|
2.24	What type of soap was used?	01….None<br>02….Local soap bar<br>03….Detergent powder<br>04….Mix (bar and detergent)<br>05….Bleach<br>99….Don't know	|__|__|	|__|__|	|__|__|
2.25	Was the net scrubbed hard or beaten on a hard surface?	01….Yes<br>00….No<br>99….Don't know	|__|__|	|__|__|	|__|__|

2.26	Where was the net dried?	01….Outside in the direct sun light<br><br>02….Outside in the shade<br><br>03….Inside<br><br>99….Don't know	|__|__|	|__|__|	|__|__|
2.27	Have you tried to fix any of holes in this net?	01….Yes<br><br>00….No, **go to 2.29**	|__|__|	|__|__|	|__|__|
2.28	How did you repair the hole?<br><br>**Go to 2.30**	01…Stitched<br><br>02…Knotted/tied<br><br>03…Patched<br><br>04…Other way, specify	|__|__| <br>__________	|__|__| <br>__________	|__|__| <br>__________
2.29	If not, what was the main reason?	01…Too busy/no time<br><br>02…Not necessary<br><br>03…Don't know how to fix<br><br>04…Too damaged to fix<br><br>05…Other, specify	|__|__| <br>__________	|__|__| <br>__________	|__|__| <br>__________
2.30	Has the net been modified?	01…Yes<br><br>00…No, **go to Section 3**	|__|__|	|__|__|	|__|__|
2.31	How was the net modified?	01…Shape was changed<br><br>02…Material was added to lengthen<br><br>03…Material was added to reinforce<br><br>04…Other, specify	|__|__| <br>__________	|__|__| <br>__________	|__|__| <br>__________
Section 3<br>***"I am going to read a series of statements to you and I would like you to tell me how much you agree with them"***					
3.1	Which of these statements does best describe your net?	01… This net is still in a good condition and can be used without restrictions<br><br>02… This net is beginning to fall apart and should be replaced really soon<br><br>03… This net is no longer usable and definitely needs to be replaced	|__|__|	|__|__|	|__|__|
**Section 4 Net inspection**<br>***"Now I will have a look at your nets and count the number of holes. The net will be returned to you and hung up again if you wish. We need to mount the net on a frame in order to find all the holes."***					

Interviewer to mount net 1 on net frame for hole counting. Make sure that only one net is done at a time and enter the data directly from tally sheet into the PDA.

<table>
<tr><td>4.1</td><td>What type of holes are observed?

Answer every category</td><td>01…Yes

00…No</td><td>No holes – if 01, go to Section 5</td><td>|__|__|</td><td>|__|__|</td><td>|__|__|</td></tr>
<tr><td></td><td></td><td></td><td>Horizontal tears at bottom</td><td>|__|__|</td><td>|__|__|</td><td>|__|__|</td></tr>
<tr><td></td><td></td><td></td><td>Holes at hanging points</td><td>|__|__|</td><td>|__|__|</td><td>|__|__|</td></tr>
<tr><td></td><td></td><td></td><td>Open seams</td><td>|__|__|</td><td>|__|__|</td><td>|__|__|</td></tr>
<tr><td></td><td></td><td></td><td>Burn holes</td><td>|__|__|</td><td>|__|__|</td><td>|__|__|</td></tr>
<tr><td></td><td></td><td></td><td>Holes from rodents</td><td>|__|__|</td><td>|__|__|</td><td>|__|__|</td></tr>
<tr><td></td><td></td><td></td><td>Whole section missing</td><td>|__|__|</td><td>|__|__|</td><td>|__|__|</td></tr>
<tr><td></td><td></td><td></td><td></td><td>|__|__|</td><td>|__|__|</td><td>|__|__|</td></tr>
<tr><td>4.2</td><td>Number of holes in zone 1</td><td colspan="2">Size 1 (finger)</td><td>|__|__|</td><td>|__|__|</td><td>|__|__|</td></tr>
<tr><td></td><td></td><td colspan="2">Size 2 (fist)</td><td>|__|__|</td><td>|__|__|</td><td>|__|__|</td></tr>
<tr><td></td><td></td><td colspan="2">Size 3 (head)</td><td>|__|__|</td><td>|__|__|</td><td>|__|__|</td></tr>
<tr><td></td><td></td><td colspan="2">Size 4 (larger than head)</td><td>|__|__|</td><td>|__|__|</td><td>|__|__|</td></tr>
<tr><td>4.3</td><td>Number of holes in zone 2</td><td colspan="2">Size 1 (finger)</td><td>|__|__|</td><td>|__|__|</td><td>|__|__|</td></tr>
<tr><td></td><td></td><td colspan="2">Size 2 (fist)</td><td>|__|__|</td><td>|__|__|</td><td>|__|__|</td></tr>
<tr><td></td><td></td><td colspan="2">Size 3 (head)</td><td>|__|__|</td><td>|__|__|</td><td>|__|__|</td></tr>
<tr><td></td><td></td><td colspan="2">Size 4 (larger than head)</td><td>|__|__|</td><td>|__|__|</td><td>|__|__|</td></tr>
<tr><td>4.4</td><td>Number of holes in zone 3</td><td colspan="2">Size 1 (finger)</td><td>|__|__|</td><td>|__|__|</td><td>|__|__|</td></tr>
<tr><td></td><td></td><td colspan="2">Size 2 (fist)</td><td>|__|__|</td><td>|__|__|</td><td>|__|__|</td></tr>
<tr><td></td><td></td><td colspan="2">Size 3 (head)</td><td>|__|__|</td><td>|__|__|</td><td>|__|__|</td></tr>
<tr><td></td><td></td><td colspan="2">Size 4 (larger than head)</td><td>|__|__|</td><td>|__|__|</td><td>|__|__|</td></tr>
<tr><td>4.5</td><td>Number of holes in zone 4</td><td colspan="2">Size 1 (finger)</td><td>|__|__|</td><td>|__|__|</td><td>|__|__|</td></tr>
<tr><td></td><td></td><td colspan="2">Size 2 (fist)</td><td>|__|__|</td><td>|__|__|</td><td>|__|__|</td></tr>
<tr><td></td><td></td><td colspan="2">Size 3 (head)</td><td>|__|__|</td><td>|__|__|</td><td>|__|__|</td></tr>
<tr><td></td><td></td><td colspan="2">Size 4 (larger than head)</td><td>|__|__|</td><td>|__|__|</td><td>|__|__|</td></tr>
<tr><td>4.6</td><td>Number of holes in the roof</td><td colspan="2">Size 1 (finger)</td><td>|__|__|</td><td>|__|__|</td><td>|__|__|</td></tr>
<tr><td></td><td></td><td colspan="2">Size 2 (fist)</td><td>|__|__|</td><td>|__|__|</td><td>|__|__|</td></tr>
</table>

		Size 3 (head)<br><br>Size 4 (larger than head)	|__|__| <br>|__|__|	|__|__| <br>|__|__|	|__|__| <br>|__|__|
**Section 5 Additional Nets In Household**<br><br>***"This part is about any additional nets apart from the ones you received from our study team last October you may have inside your household. Please could you show us the nets and spare some time to answer the subsequent questions."***					
5.1	Do you own any additional nets in addition to the ones distributed by our study team?	01… Yes<br><br>00… No, **Go to NEXT SECTION**	|__|__|		
5.2	How many additional nets do you have?	***Enter number***	|__|__|		
			Net 1	Net 2	Net 3
5.3	Where is the net located?<br><br>***Observe, if unsure - ask***	01…Hanging loose over a sleeping space<br><br>02…Hanging and folded up or tied<br><br>03…Stored inside a bag<br><br>04…Stored but not in a bag<br><br>05…Other, specify	|__|__| <br>__________	|__|__| <br>__________	|__|__| <br>__________
5.4	What type of sleeping space is the net used for?<br><br>***Observe, if unsure - ask***	01…Wooden bedframe<br><br>02…Stick bedframe<br><br>03…Iron bedframe<br><br>04…Mattress (no frame)<br><br>05…Reed mat (no frame)<br><br>06…Floor<br><br>07…Never used → **go to 5.5**<br><br>08…Other, specify	|__|__| <br>__________	|__|__| <br>__________	|__|__| <br>__________
5.5	Why has the net never been used?<br><br>**Go to 5.11**	01...Save the net for visitors<br><br>02…Save the net for future use<br><br>03…No place to hang up<br><br>04…Currently have enough nets in use<br><br>05…Other, specify	|__|__| <br>__________	|__|__| <br>__________	|__|__| <br>__________

5.6	Was this net used last night?	01… Yes, **Go to 5.8**<br><br>00… No	|__|__|	|__|__|	|__|__|
5.7	If not, why not?<br><br>***Do not prompt. Record all reasons that the person mentions.***<br><br>**Go to 5.9**	01…User did not sleep here<br>02…Net was not available to use<br>03…Used a different net<br>04…No malaria now<br>05…No mosquitoes<br>06…Net too old or too torn<br>07…Net is dirty<br>08…Net full of bedbugs<br>09…Net too hot<br>10…Net too small<br>11…Net too big<br>12…Mesh size too big<br>13…Don't like the material<br>14…Don't like the colour<br>15… Don't like the smell<br>16…Net made me sneeze, itch, head ache<br>17…Other, specify<br>99…Don't know	|__|__| __________	|__|__| __________	|__|__| __________
5.8	Who used this net last night?	***Drop down menu with names from household roster 1.2. Follow up with "Is [name] x years old?"***<br><br>***Allow multiple choices***	__________<br>__________<br>__________<br>__________	__________<br>__________<br>__________<br>__________	__________<br>__________<br>__________<br>__________
5.9	During the previous week, how many times has the net been used?	01…Every night<br>02…5-6 nights<br>03…1-4 nights<br>04…None of the nights	|__|__|	|__|__|	|__|__|

		99…Don't know			
5.10	How long ago did you start using this net?	01…Less than 1 week ago<br>02…Between 1 week and 1 month ago<br>03…Between 1-6 months ago<br>04…Between 6-12 months ago<br>05…More than 1 year ago<br>06…Never used<br>99...Don't know	|__|__|	|__|__|	|__|__|
5.11	What is the colour of the net?<br>***Observe***	01…White<br>02…Light blue<br>03…Blue & white stripes<br>04…Dark blue<br>05…Green<br>06…Other	|__|__|	|__|__|	|__|__|
5.12	What is the shape of the net?	01…Round<br>02…Rectangular	|__|__|	|__|__|	|__|__|
5.13	What is the size of the net?	01…Single<br>02…Double<br>03…Extra-large	|__|__|	|__|__|	|__|__|
5.14	What is the brand of the net?<br>***Check label if present***	01…Olyset / A-Z / Sumitomo<br>02…Safinet<br>03…PermaNet / Vestergaard Frandsen<br>04…Netprotect / BestNet<br>05…Interceptor / BASF<br>06…LifeNet / Bayer<br>07…Yorkool	|__|__| __________	|__|__| __________	|__|__| __________

		08…DawaPlus / Tana Netting<br><br>09…Duranet / Clarke<br><br>10…Royal Sentry<br><br>11…MAGNet<br><br>12…Afyanet<br><br>13…Health net Ltd / Net health Ltd<br><br>14…Other, specify<br><br>99..Don't know, no label			
5.15	How long ago did you obtain this net?	01…Less than 1 week ago<br><br>02…Between 1 week and 1 month ago<br><br>03…Between 1-6 months ago<br><br>04…Between 6-12 months ago<br><br>05…More than 1 year ago<br><br>99...Don't know	|__|__|	|__|__|	|__|__|
5.16	Where did you obtain this net from?	01…Received as a gift from relative /friend/neighbour<br><br>02…Bought in shop/market<br><br>03…Received from hospital/dispensary<br><br>04…Received from NGO/charity<br><br>05…Received from government campaign<br><br>06…Other, specify<br><br>99…Don't know	|__|__| <br>__________	|__|__| <br>__________	|__|__| <br>_________
5.17	Did you pay money for this net?	01…Yes<br><br>00…No<br><br>99…Don't know	|__|__|	|__|__|	|__|__|
5. 18	Did you use a voucher to obtain this net?	01…Yes<br><br>00…No	|__|__|	|__|__|	|__|__|

		99…Don't know				
5.19	Does the net have any open holes/tears/seams?<br><br>***Observe inside the house***	01…Yes<br><br>00…No, **Go to 5.22**		|__|__|	|__|__|	|__|__|
5.20	What type of holes are observed?<br><br>***Observe inside the house***<br><br>***Answer every category***	01…Yes<br><br>00…No	Horizontal tears at bottom<br><br>Holes at hanging points<br><br>Open seams<br><br>Burn holes<br><br>Holes from rodents<br><br>Whole section missing	|__|__| |__|__| |__|__| |__|__| |__|__| |__|__| |__|__|	|__|__| |__|__| |__|__| |__|__| |__|__| |__|__| |__|__|	|__|__| |__|__| |__|__| |__|__| |__|__| |__|__| |__|__|
5.21	Is there any evidence of repair of the net?<br><br>***Observe inside the house***	01…Yes<br><br>00…No		|__|__|	|__|__|	|__|__|

************** END OF THE QUESTIONNAIRE **************

Please write whether there were any comments about the study or the nets / any messages from the households.

…………………………………………………………………………………………………......

…………………………………………………………………………………………………......
